# Physiological and Medical Researches into the Causes, Symptoms, and Treatment of Gravel

**Published:** 1819-10-01

**Authors:** 


### XIII.

*Physiological and Medical Researches into the Causes, Symptoms, and Treatment of Gravel.* By F. Magendie, M. D. One Vol. small 8vo. pp. 103. London, 1818.

Many of our readers may have perused this little work, but the majority of them have not; and we must be mindful of all. Our labours are not directed to the gratification of the learned, by *early* presenting every *novelty* in medical literature, but to the improvement of the *humble*, by bringing within their reach, a clear and steady stream of useful and practical lore. The Medico-Chirurgical

Journal is not an Ephemeral Butterfly that flirts from flower to flower; but the provident Bee, that lays up the honey in well constructed combs, where it is always ready for use. In short, it is more ambitious of becoming a *reference* at the *end* of the year, than a subject of eager perusal at the *beginning* of the quarter. Happy should we be, however, if we could unite in it both these attractive qualities!

The *symptoms* of gravel are too well known to be described here; but the nature, cause, and treatment of the complaint, may well occupy a few pages. The discovery of Scheele, that urinary concretions were almost entirely composed of uric acid, united with a small proportion of animal matter, has been confirmed by modern chemists. The cases where gravel has been found, wholly, or in part, composed of oxalate and phosphate of lime, cystic oxyde, &c. are no more than exceptions to general rules, and need not occupy our attention in this place.

The urine of man, and of all animals that feed principally on flesh, fish, eggs, &c. containing a considerable proportion of azote, abounds with *uric acid*. If, on the other hand, animals feed exclusively on vegetables, the urine furnishes no trace of the said acid. M. Magendie, from a series of experiments, discovered, that if carnivorous animals be deprived of all food containing azote, and fed on sugar, gum, oil, and the like, their urine, in three or four weeks, will become entirely devoid of uric acid.

This substance, when in a state of purity, is solid; pale yellow; heavier than water; without taste or odour; undecomposable by the air, and requires nearly 1800 times its own weight of water, at 65° or 70° to dissolve it. It is soluble in 1200 times its own weight of *boiling* water, the superabundance depositing, on cooling, in the form of small flakes. Nearly all the acids decompose it.

M. Magendie thinks, then, that there are three evident causes of gravel.

"1°. An increase of the uric acid, the urine remaining the same.

"2°. A diminution of the urine, the acid remaining the same.

"3°. A diminution of the temperature of the urine."

He is far from thinking, however, that these are the *only* causes, although he lays little or no stress on the functional derangements of the chylo-poietic viscera, as causes of the complaint—a great defect in our author's work—his ideas being too chemical or mechanical. We entirely agree, however, with our author, in the following passage.

"In the first rank of causes which increase the proportion of uric acid in the urine, and which consequently often produce gravel, are, very nutritious diet, an habitual use of sumptuous tables, with highly flavoured dishes, particularly those prepared with animal substances; in a word, the diet of the rich, and of the amateurs of good living."

Dr. M. exemplifies this by the case of a Hanse merchant, who went through several vicissitudes of fortune, and also of health. When rich, he gormandized, and had the gravel; when reduced to poverty, he lived abstemiously, *par force*, and got clear of his complaint. When raised again to affluence, he resumed his habits, and with them the gravel. Now, in our minds, these examples prove nothing towards the Doctor's theory; but, on the contrary, support the opinions of the British practitioners, who look upon imperfect digestion and assimilation, with their consequences, *deranged secretions*, as the great operative causes of depositions in the *urine*, as well as morbid states of the *fæcal* evacuations. This last circumstance is passed unnoticed by the French physician, though we have seen it so very generally co-existing with the uric acid, as to leave no doubt that they both proceeded from one cause, over-exertion and stimulation of the digestive organs. Nor is this doctrine incompatible with the observation of Dr. Magendie, that sedentary habits and advanced age, when combined with full diet, contribute strongly to the production of gravel. But although we conceive Dr. M's theory to be, at least, defective, his practice is very well worth attending to.

It has been stated, that a diminution of the aqueous part of the urine is favourable to the deposition of uric acid. Thus beer, cyder, and other drinks, composed principally of water, have no tendency towards the generation of gravel, whereas the contrary is the case, where wines and alcoholic liquors are used; a confirmation of which remark will be found in page 107 of this volume. Abundant cutaneous perspiration, profuse fluid evacuations, &c. will be favourable to the formation of gravel, by furnishing obstacles to the solution of the uric acid. The same may be said of the habit of lying long in bed, which diminishes the urinary and augments the perspiratory process.

Respecting the influence of the *temperature* of the urine on the formation of gravel, we need say little; for although there can be no doubt that our youthful blood flows warmer in our veins, than when TIME has chilled the rosy current, and strewed the hoary frost of age on our heads—

> ——— Cum gelidus, tardante senecta,

> Sanguis hebet, frigentque effætæ in corpore vires,

Yet we much doubt whether the old man's calculous concretions be owing to mere *diminution* of temperature. Nature appears, at that time, to be silently and gradually converting the material fabric into clay, preparatory to the final departure of, we hope, its immaterial tenant; and therefore this increased disposition to the formation of uric acid is but one of the many indications of an approaching affinity for the grave yard!

Neither is it our intention to notice the many fanciful and absurd theories, respecting the cause or the prevention of gravel, which have been entertained by authors and visionaries. M. Magendie combats the idea entertained by many writers, that muriate of soda, or culinary salt, occasions gravel. By this time he has heard of an opposite theory lately started, that this *same salt* prevents the formation of uric acid, and gives sailors an immunity from stone, in recompence for the severity of their *salt junk fare!*

*Treatment*. The *first* indication of our author is to restrict the diet of animal food, substituting a vegetable one for it. When patients adopt this regimen they must avoid spirituous liquors, and wine in an undiluted state, drinking, at the same time, copiously of aqueous fluids, which answers the *second* indication, namely, that of increasing the quantity of urine.

The *third* indication is to saturate the uric acid with the carbonate of potash or soda; but these, Dr. M. thinks should not be carried farther than from 24 to 36 grains in the day, otherwise the functions of the stomach are liable to derangement. Where pure potash and soda are administered, there must still be greater precaution. They should be so diluted as hardly to leave any taste upon the tongue. A pint of such solution may be taken daily. Magnesia, as recommended by Mr. Brande, may also be employed. Dr. Magendie has never been able to put a stop to the alkalescency of the urine by the administration of the mineral or vegetable acids. On the other hand, he has seen the most violent paroxysm of nephritic pains, from calculi, alleviated in a few hours by the alkalies. These medicines, however, as he justly observes, are mere palliatives, unless the regimen be changed, and the *fons et origo mali* attacked.

We have hinted our opinion, that Dr. Magendie's chemical theory was an illusion, and that the success of his *regimen* depended solely or almost entirely on the improvement of the digestive functions which resulted. The following passage from himself will corroborate our opinion.

"The greater number of patients, for example, derive benefit from the means proper to relieve *dyspepsia*, which often accompanies the disease; and among those means, magnesia, in small doses, rhubarb, cinchona, and sulphurous waters, taken internally, are frequently attended with success. *I have seen repeated purgatives, administered so as to produce considerable evacuations, have the most fortunate results. In this, as in many other respects, gravel somewhat resembles gout, rheumatism, dropsy, and other chronic maladies, which are often alleviated by the same measures.*" P. 98.

This passage speaks for itself, and, as our author has candidly acknowledged, "theory becomes silent in the presence of experience."

---

The following observations on the MEDICO-CHEMICAL treatment of calculous disorders, principally analysed from Mr. Brande's paper on the subject, will appropriately conclude this short Review of Magendie.

The chemical physicians are very proud of the successful application of chemical science to the practice of medicine, in this instance; yet Mr. Brande [*Jour. of Arts,* xii] is forced to acknowledge, that "there are very few cases in which chemical principles are successfully applicable to the treatment of disease." Every physician who looks beyond the surface of things will bear witness, that the chemical agents employed for neutralizing the acids or alkalis, which appear in the urine, are little more than placebos. They merely obviate *effects*—they remove not the *causes* of disease.

The principal deposits in human urine are, phosphate of lime, phos. magnes. vel ammon. and uric acid. The two first constitute a *white*, the last a *red* deposit. The *white*, as well as the *red* sand, is almost always symptomatic of disordered digestion, and deranged biliary secretion, especially where excess in eating or drinking has been committed. It *seems* to be produced by the inordinate use of farinaceous diet or alkaline medicines; magnesia, and soda water will produce it in some constitutions. If the *white* sand invariably follow meals, and appear in the urine even before cooling, there is danger of *calculous* concretions, besides irritation of the urinary apparatus. Where the mineral acids do not disagree, they will check the formation of the white sand; but where they irritate the stomach, bowels, or bladder, the vegetable and especially the citric acid, in doses of from five grains to half a drachm, is very effectual. But often we find accompanying this deposit, an irregular secretion of bile, pain in the region of the liver, sallow complexion, whitish brown and dry tongue, &c. with deranged functions of the intestines. Here an acescent diet, with gentle laxatives, as a tea spoon full of magnesia in a glass of sour lemonade, in the morning, with plenty of oranges, will have a good effect. The biliary secretion must also be improved by alterative doses of the blue pill, and bitter infusions. The carbonic acid is strongly recommended by Professor Brande, in the form of an effervescing draught [xxx gr. carb. potass. xx gr. citric acid, dissolved separately, and drunk immediately on commixture] two or three times a day. The febrile affections of children are very frequently attended by a copious deposit of white sand, and both will be carried off by a dose or two of calomel.

When the *red sand* falls to the bottom of the urine, when fresh made, it indicates an alarming tendency to form calculi; where it separates from the urine on cooling, there is much less danger. As a corrector of this state, *soda water*, Mr. Brande thinks, upon the whole, the best medicine. But it must be good—not merely water impregnated with fixed air. This medicine, however, is sometimes ineffectual. The solution of potash is then to be employed, or ammonia, and subcarbonate of ammonia. Where these disagree, magnesia will be found a valuable succedaneum. From ten to twenty drops of the liquor potassæ may be considered an average dose, taken night and morning. Subcarbonate of ammonia made into pills, with extract of chamomile [subcar. ammon. ℈j. ex. chamom. ʒj, in xxiv pills] is an excellent remedy. Two or three of the above pills twice or thrice a day.

In all these cases, however, the abdominal secretions must be restored to a healthy state, otherwise the labour of correcting the urinary aberrations, is like that of Sisyphus—the *stone* is ever rolling back upon us!

